# Damage Assessment of Two-Way Bending RC Slabs Subjected to Blast Loadings

**DOI:** 10.1155/2014/718702

**Published:** 2014-07-08

**Authors:** Haokai Jia, Ling Yu, Guiying Wu

**Affiliations:** ^1^Department of Mechanics and Civil Engineering, Jinan University, Guangzhou 510632, China; ^2^MOE Key Laboratory of Disaster Forecast and Control in Engineering, Jinan University, Guangzhou 510632, China; ^3^College of Mechanics, Taiyuan University of Technology, Taiyuan, Shanxi 030024, China

## Abstract

Terrorist attacks on vulnerable structures and their individual structural members may cause considerable damage and loss of life. However, the research work on response and damage analysis of single structural components, for example, a slab to blast loadings, is limited in the literature and this is necessary for assessing its vulnerability. This study investigates the blast response and damage assessment of a two-way bending reinforced concrete (RC) slab subjected to blast loadings. Numerical modeling and analysis are carried out using the commercial finite element code LS-DYNA 971. A damage assessment criterion for the two-way bending RC slab is defined based on the original and residual uniformly distributed load-carrying capacity. Parametric studies are carried out to investigate the effects of explosive weight and explosive position on the damage mode of the two-way RC slab. Some design parameters, such as the boundary conditions and the negative reinforcement steel bar length, are also discussed. The illustrated results show that the proposed criterion can apply to all failure modes. The damage assessment results are more accurate than the ones due to the conventional deformation criterion.

## 1. Introduction

Over the last two decades, increased terrorist attacks on civilian and military structures have drawn considerably more attention to the vulnerability and sustainability of structures and their individual structural members when subjected to blast loadings [1]. However, the research work on response and damage analysis of single structural components, for example, a slab to blast loadings, is limited in the literature and this is necessary for assessing its vulnerability. Some investigations have been conducted mainly for one-way slab based on one of the three methods: (1) theoretical analysis by equivalent single degree of freedom (SDOF) systems, (2) finite element analysis methods, and (3) the field measurements. Two loosely coupled SDOF systems were used to analyze the failure model of one-way bending RC slab subjected to blast loadings, and the results showed that the failure mode of slab tends to be direct failure [2]. Jones et al. developed one finite difference program to simulate the dynamic response of a simply supported RC slab under blast loadings, and the deformation of slab was due to the duration time and peak value of blast loadings [3]. However the SDOF model cannot forecast the localized damage element of the structure; more and more studies are based on commercial software, such as LS-DYNA, AUTODYN, and ABAQUS. Xu and Lu used LS-DYNA set up a three-dimensional RC plate to simulate the concrete material spallation under blast loadings [4]. Zhou et al. used a dynamic plastic model with the software AUTODYN and found that the erosion technique can be used to evaluate concrete spall together with the damage scalar [5, 6]. Tai et al. used the erosion technique to analyze dynamic response of RC slab under the air blast loadings, and the results clearly showed the damage modes by the software LS-DYNA [7].

At the same time, the experiments of RC slab subjected to blast loadings were conducted by some researchers. Wu et al. have designed series of experiments of unretrofitted and retrofitted RC slabs under blast loadings to find the rule of fragment size and shape [8]. Schenker et al. illustrated the dynamic responses of unprotected and protected RC slabs subjected to blast loadings and compared the results with numerical simulation by MSC Software/Dytran [9]. Chung and Nurick analyzed the response of quadrangular stiffened plates under explosion by experiments and numerical simulation [10, 11]. Wang et al. have performed blast tests and numerical simulation, respectively, to investigate the damage modes of one-way square RC slab subjected to closing blast loadings [12, 13]. They defined the damage degree of the slab with the support rotation. Silva and Lu proposed a step-by-step method for measuring the damage degree level of RC slabs under explosion by experiments [14].

However the above studies focused on the dynamic responses and the failure models of the RC slab subjected to blast loadings. In references [12–14] the damage degree assessment was defined with the deformation of slabs as the support rotation, but it was not satisfied to all failure modes and it could be used for closing blast loads. Shi et al. [15] and Wu et al. [16] used the residual axial load-carrying capacity and axial compression capacity to assess the damage degree of RC columns subjected to blast loadings, and they concluded that the assessment contrition can be applied to all failure modes of columns, but they did not report if it is suitable for the other individual structural members, for example, the slabs.

Based on the residual uniformly distributed load-carry capacity, an assessment criterion is proposed for evaluating the damage of a two-way bending RC slab subjected to blast loadings in this study. It is defined with the decrease range of load-carry capacity. Numerical analysis and damage assessment are carried out by using the commercial finite element code LS-DYNA 971. The effects of explosive weight and position on the damage situation are studied. Some design parameters, such as the boundary conditions and the negative reinforcement steel bar length, are discussed as well. The illustrated results show that the criterion can evaluate the damage more accurately and it can be applied to all failure modes of structures.

## 2. Geometry Model and Material Properties

### 2.1. Geometrical Modeling

The most acceptable modeling methods of RC structures are the smeared model, embedded model, and discrete model [17]. It was stated in [17] that, for the dynamic response of structural components, the discrete model was the best method. In a discrete model, the concrete element and steel bar element are treated as different elements. A one-dimensional slide contact is added between the concrete and reinforcement elements to simulate the longitudinal shear behavior, and the differences in two material mechanical properties can be shown clearly when the blast loadings affect the structure.

The mechanical behaviors of the slab depend on the aspect ratio of length to width. When the aspect ratio is greater than 3, the slab can be deemed as a one-direction bending type structure, for example, a beam. When the aspect ratio is less than 3, the mechanical behavior of the slab is two-way bending. The one-way bending slabs are used mostly in corridor part, and the two-way bending slabs are used in the important components of structures, such as floors and walls.

**Figure 1:**
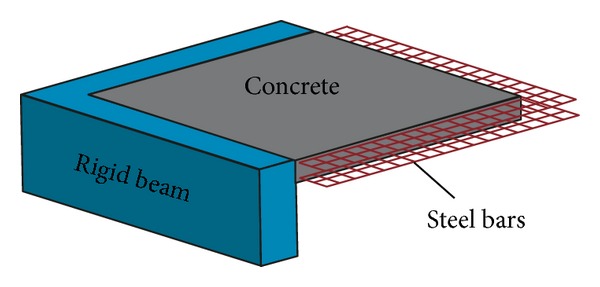
Numerical model of RC two-way bending slabs in a quarter form.

In this study, a square RC slab model is established. The clear dimension of the slab is *B* × *L* × *t* = 3000 mm × 3000 mm × 120 mm, where *B* is the slab span length in *x* direction, *L* is the slab width in *y* direction, and *t* is the slab thickness in *z* direction, respectively. The blast loadings are applied to slab in *z* direction. So the aspect ratio of length to width is 1.0, which means that the slab is a two-way bending one. The steel bar material is HPB300 class, in which the steel bar diameter is 12 mm. The concrete material is C30 class with a cube compressive strength of 30 MPa. The concrete cover thickness is 14 mm. The steel bars are layout in two-ways with two layers; the spacing between two bars in *x* direction is 100 mm and the spacing in *y* direction is also 100 mm. The mesh generation of the concrete and steel bar is 20 mm, and between the concrete and steel bar nodes there is one-dimensional slide contact. The element type of the concrete chosen is* Solid 164*, and the steel bar is* Beam 161*. The numerical model of RC slab is shown in Figure 1.

**Figure 2:**
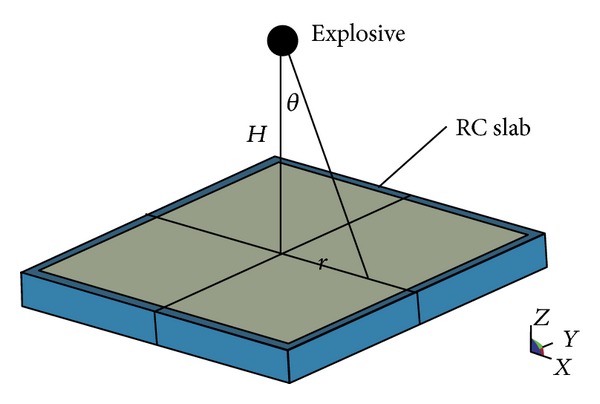
Calculating diagram of RC slab under explosion.

In order to simulate the support role of the beam to the slab, four rigid beams are set up around the slab and their connections are rigid. The explosive is on the top of slabs. The whole model is shown in Figure 2.

### 2.2. Material Models

**Figure 3:**
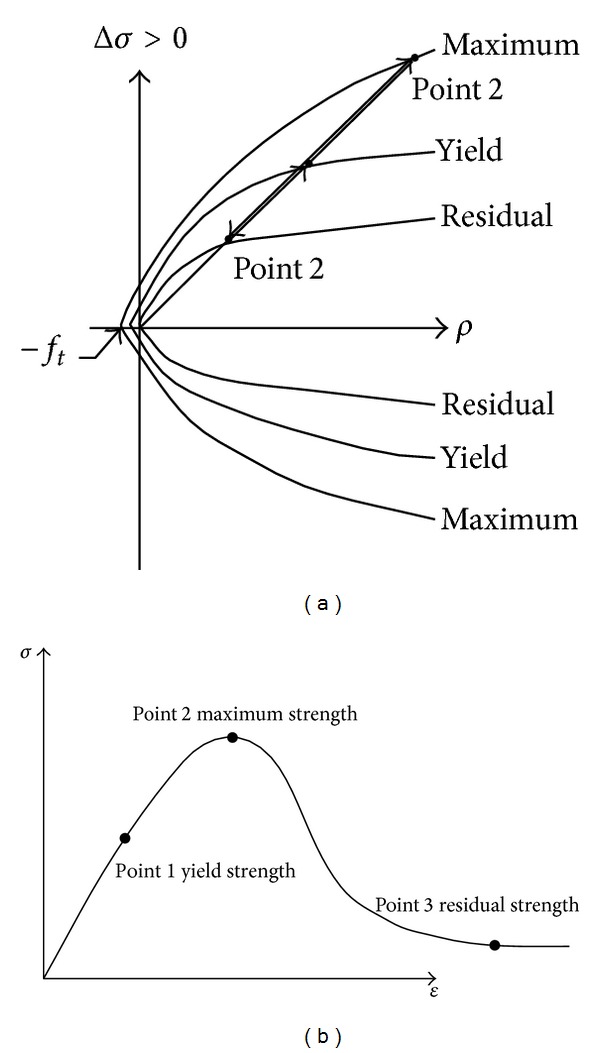
Concrete material model: (a) three strength failure surface and (b) stress-strain curve.

There are many material models to simulate concrete, such as HJC, RHT, and K&C models [18–22]. However, it is well known that the FE simulation results are very sensitive to the material properties, and thus it is the most important issues to choose the most suitable material model. The K&C model is a three-invariant model and three shear yield faces are used (Figure 3). It can simulate the mechanical behavior of concrete subject to high strain rate and large deformation [18, 19] and can simulate some concrete structure behavior under blast loadings in some cases [15, 16, 23]. The advantage of the model is that it needs very few parameters to import in LS-DYNA, and the material type corresponding to K&C model is Mat_Concrete_Damge_ Rel3 (Mat type 72 rel3). In this study, only the density, uniaxial compressive strength, and Poisson ratio are imported [24, 25]. The density of C30 concrete is 2500 kg/m^3^, uniaxial compressive strength is 20.06 MPa, and the Poisson ratio is 0.2.

Mat-Plastic-Kinematic (Mat type 3) is an isotropic and kinematic hardening plasticity model. The strain rate effect is considered using the Cowper-Symonds model, and the yield stress is defined with the following factor [24, 25]:

(1) $$\begin{array}{c} \sigma_{y}=\left[1+{\left(\frac{\dot{\varepsilon }}{C}\right)}^{1/p}\right]\left(\sigma_{0}+\beta E_{p}\varepsilon_{p}^{\text{eff}}\right), \end{array}$$

where *σ*_0_ is the yield stress under static loads, $\dot{\varepsilon }$ is the strain rate, *E*_*p*_ is the plastic hardening modulus, and *ε*_*p*_^eff^ is the effective plastic strain. *C* and *p* are the strain rate parameters; in this study, *C* = 40 s^−1^ and *p* = 5. And *β* is the hardening parameters; when *β* = 0, it means that the material is kinematic. The density of steel bar is 7800 kg/m^3^, the yield stress *σ*_0_ = 300 MPa, Young's modulus *E* = 2.1 × 10^5^ MPa, and Poisson ratio *ν* = 0.3, respectively.

### 2.3. Strain-Rate Effects

When the structures are under blast load, it will respond at very high strain rates in the order of 10–1000 s^−1^ or even higher [26]. Due to the high strain rate, the tensile and compressive ability of concrete will be changed. It needs to consider the strain-rate effects of concrete in a reliable simulation of RC structural dynamic response.

The effect of strain rate on the concrete compressive and tensile strength is typically represented by a parameter, namely, the dynamic increase factor (DIF). It is a ratio of the dynamic-to-static material constants versus strain rate. In this study the dynamic increase factor of compressive strength (CDIF) is adopted, which is recommended by Europe Code CEB [27] and defined as follows:

(2) CDIF=fdcfc={(ε.ε.stat)1.026αwhere  ε.stat < ε. <30 s−1γ(ε.ε.stat)1/3where  30 s−1< ε. <300 s−1,

where *f*_*dc*_ and *f*_*c*_ are the compressive strength with the dynamic strain rate $\dot{\varepsilon }$ and static strain rate ${\dot{\varepsilon }}_{\text{stat}}$, respectively. Here, ${\dot{\varepsilon }}_{\text{stat}}=30\;\times \;10^{-6}{\text{s}}^{-1}$, log⁡*γ* = 6.156*α* − 2, and *α* = (5 + 9*f*_*c*_/10)^−1^.

The dynamic increase factor of tensile strength (TDIF) is recommended by the corrected value due to Malvar and Ross's work [26]. It is defined as follows:

(3) $$\begin{array}{c} \text{TDIF}=\frac{f_{dt}}{f_{t}}=\{\begin{array}{cc} {\left(\frac{\dot{\varepsilon }}{{\dot{\varepsilon }}_{\text{stat}}}\right)}^{\delta } & \text{where}\;\;{\dot{\varepsilon }}_{\text{stat}}\;<\;\dot{\varepsilon }\;<1\;{\text{s}}^{-1}\\ \beta {\left(\frac{\dot{\varepsilon }}{{\dot{\varepsilon }}_{\text{stat}}}\right)}^{1/3} & \text{where}\;1\;{\text{s}}^{-1}\;<\;\dot{\varepsilon }\;<\;160{\;\text{s}}^{-1}, \end{array} \end{array}$$

where log⁡*β* = 6*δ* − 2, *δ* = 1/(1 + 8*f*_*c*_/10), and *f*_*dt*_ and *f*_*t*_ are the tensile strength with dynamic strain rate $\dot{\varepsilon }$ and static strain rate ${\dot{\varepsilon }}_{\text{stat}}$, respectively; here ${\dot{\varepsilon }}_{\text{stat}}=10^{-6}{\text{s}}^{-1}$. The static compressive strength should be 30 MPa to 70 MPa. The CEB concrete material has been applied to numerical simulation by many scholars [5, 6, 15, 16].

### 2.4. Blast Load

Whatever the explosion happened within or without the structure, the pressure exerting on the slab is not uniform distribution. It depends on the relative location between the explosive and the slab, the periphery structural component distribution, the direction of the shock wave motion, and so on. At the same time, the slab will be subjected to more than one impact by the reflected wave. In this study, the keyword “load_blast” [24] in the software LS-DYNA is used to define the pressure of explosive exerted on the slab, and the pressure is calculated by CONWEP [28].

In CONWEP, it requires a list of the surface segments that will experience the blast loading, the explosive weight, and explosive position. The calculation principle is based on the scale distance in Brode function. The CONWEP algorithms do account for incidence angle by combining the reflected pressure (normal-incidence) value and the incident pressure (side-on incidence) value, and the formula from TM5-1300 [29] is used in the method. The pressure on the structure is given as follows [25]:

(4) $$\begin{array}{c} P=P_{r}\cos^{2}\theta +P_{i}\left(1+\cos^{2}\theta -2\cos\theta \right), \end{array}$$

where *P* is the pressure exerted on the slab, *P*_*i*_ is the incident pressure, *P*_*r*_ is the reflected pressure, and *θ* is the incidence angle, respectively.

### 2.5. Validation of Numerical Approach

**Figure 4:**
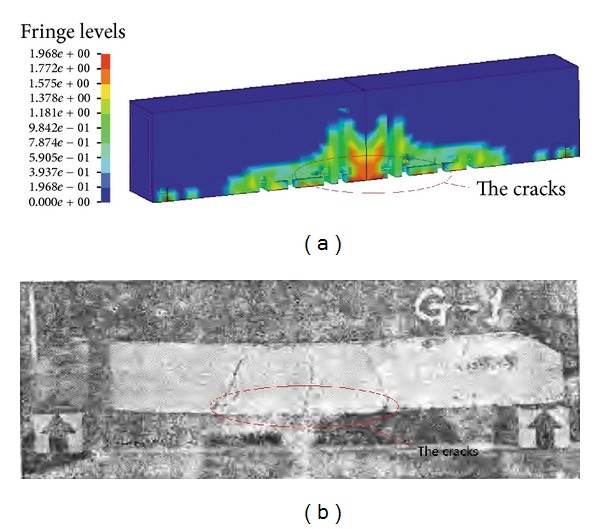
Comparison between FE model (a) and experimental model (b) of structures.

**Figure 5:**
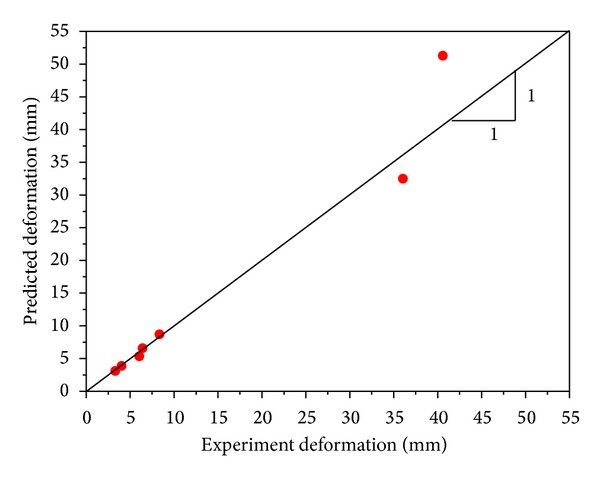
Comparison of experimental and predicted midspan deformations.

The dynamic response of RC structure components under blast loadings is tested by Antiknock studio of Tsinghua University [30]. In this study, the RC beam under blast load test is used to validate the proposed modeling method and material model chosen. A typical specimen (beam G1 in references [30]) experimental deformation is compared with the FE prediction. The cracks occurred at the bottom in the midspan area of the beam (Figure 4), and the largest displacement and the reinforcement stress are consistent with the experimental measurements. For the specimen G1, the strain of tensile reinforcement is 2.92 × 10^−3^ in the test and 2.79 × 10^−3^ in the FE calculation, the difference between the test and FE results is only 4.5%, and the tensile reinforcements are all in an elastic stage; The largest displacements in the test and FE calculation are 3.3 mm and 3.42 mm; the difference is 3.63%. For the specimen G2, the largest displacement difference is only 3.75%, and the tensile reinforcements are both yield in the test and FE calculation. The comparison on the predicted results by the FE analysis and the experimental measurements in other cases is plotted in Figure 5. It can be seen that the predicted results by FE analysis are in good agreement with the experimental observations, particularly for the deformations fewer than 10 mm.

## 3. Dynamic Behaviors of RC Slabs Subject to Blast Loadings

This section presents the dynamic responses and failure modes of RC slabs. The deformation and failure modes under blast loadings are described in Sections 3.1 and 3.2. Also the effects of explosive position (Section 3.3) are considered. Details are presented in the following sections.

### 3.1. Peak Displacement of Centre Point in Slab

Subjected to blast loadings, the most sensitive factor to the dynamic response of a structure is the peak value of blast loading. When the explosive is exploded in the air, the peak value of blast loading exerted on the slab is defined by the scaled distance. By the Brode function, if the scaled distance increases, the incident pressure will decrease and the reflected pressure will decrease too [31], so the peak value of blast will decrease. The scaled distance is defined as follows:

(5) $$\begin{array}{c} Z=\frac{H}{\sqrt[{3}]{W}}, \end{array}$$

where *Z* is the scaled distance, *W* is the explosive weight, and *H* is the distance between the explosive and the target point [28].

**Figure 6:**
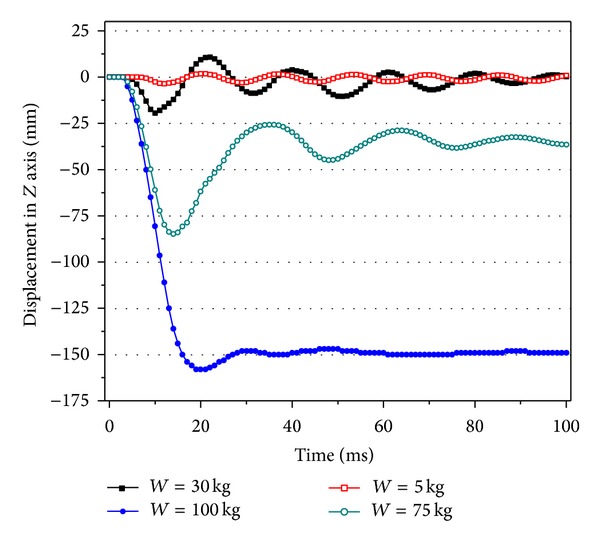
Displacement time history of RC slabs under different explosive weights.

In the simulation, the dynamic response is calculated with blast loadings from different explosive weights, and *H* is equal to 5.0 m. The results are shown in Table 1 and Figure 6.

It can be seen that the explosion happened in time *t* = 0 ms, the shockwave achieves the centre point of RC slab at 4 ms, and at the same time the slab starts to vibrate along the shockwave motion direction (the negative direction in *Z* axis). The displacement of RC slab is negative and achieves the maximum deformation rapidly. After that, the slab vibrates in a high frequency at the equilibrium position. When the explosive weight is smaller (the explosive weight is 5 kg), no plastic deformation is occurred in the slab after explosion, and the slab is in a free vibration. When the explosive weight is larger (the explosive weight is larger than 30 kg), the plastic deformation is occurred in the RC slab subjected to the blast loadings, and the slab will vibrate at the new equilibrium position. The new equilibrium position is the residual deformation of RC slabs under blast loadings.

**Figure 7:**
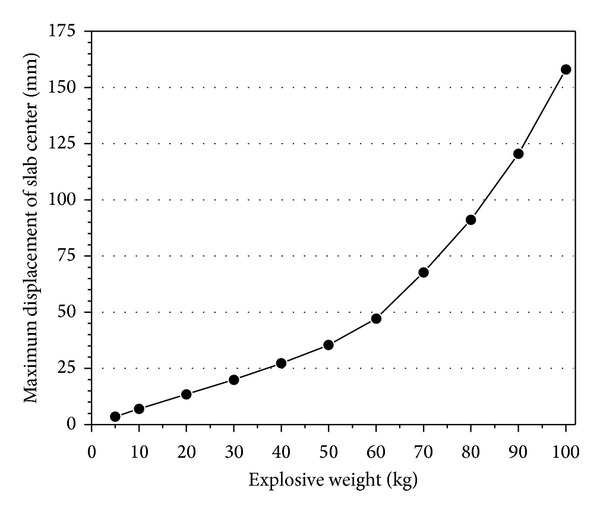
Maximum displacement of RC slabs under different explosive weights.

Figure 7 represents the relationship of maximum displacement and explosive weights. With the increase of explosive weight, the maximum displacement is increased as a curve and the slope is also increased.

### 3.2. Failure Modes

Subjected to blast loadings, the failure modes of the one-way bending RC slab are often to be one of the flexural failure, direct shear failure, and flexural-shear failure [2], respectively. The failure modes of two-way bending RC slab are investigated here.

**Figure 8:**
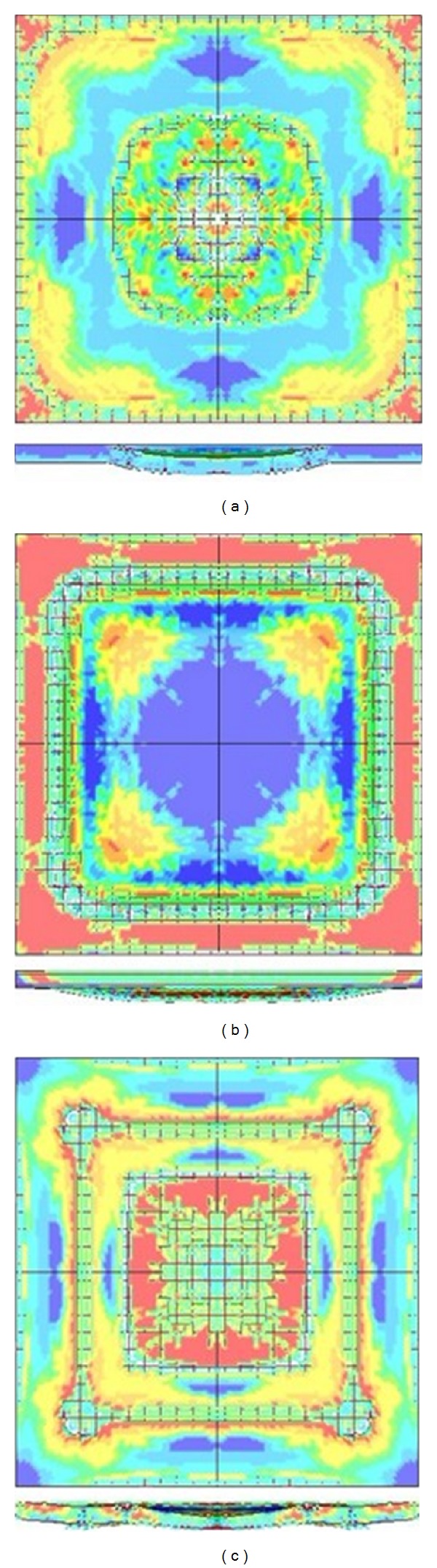
Failure modes: (a) flexural failure, (b) direct-shear failure, and (c) flexural-shear failure.

When the explosive weight *W* = 50 kg, the distance *H* = 5.0 m, and the scaled distance is 1.35 m/kg^1/3^, the peak value of blast loading exerted on the slab is *P*_max⁡_ = 2.03 MPa. The failure mode of RC slab is flexural failure. It shows that the concrete of tensile zone at midspan is destroyed and the steel bar in this zone is yielded, and even for the compressive zone located on the upper region of mid-span, it is also destroyed. The destroyed area is concentrated in the centre of slab as shown in Figure 8(a).

When the explosive weight *W* = 200 kg, the distance *H* = 5.0 m, and the scaled distance is 0.85 m/kg^1/3^, the peak value of blast loading exerted on the slab is *P*_max⁡_ = 8.14 MPa. The failure mode of RC slab is direct-shear failure. The reason is that the peak value of the blast loading is at a high level and the duration time is short, and the shear stress at the zone near to the support increased rapidly, so this zone is destroyed. At the same time, the bending deformation did not even occur. The main characteristics of the direct-shear failure mode are the destroyed area concentrated in the regions nearby the boundary as shown in Figure 8(b).

When the explosive weight *W* = 100 kg, the distance *H* = 5.0 m, and the scaled distance is 1.08 m/kg^1/3^, the peak value of blast loading exerted on the slab is *P*_max⁡_ = 4.02 MPa. The failure mode of RC slab is flexural-shear failure. As the *P*_max⁡_ is larger than the load which caused flexural failure and it is smaller than the load which caused direct-shear failure, there are destroyed areas in both the centre and the boundary as shown in Figure 8(c).

The failure mode of RC slab will appear in the form of flexural failure, flexural-shear failure, and direct-shear failure sequentially when the explosive weight is increased.

### 3.3. Effect of Explosive Position

**Figure 9:**
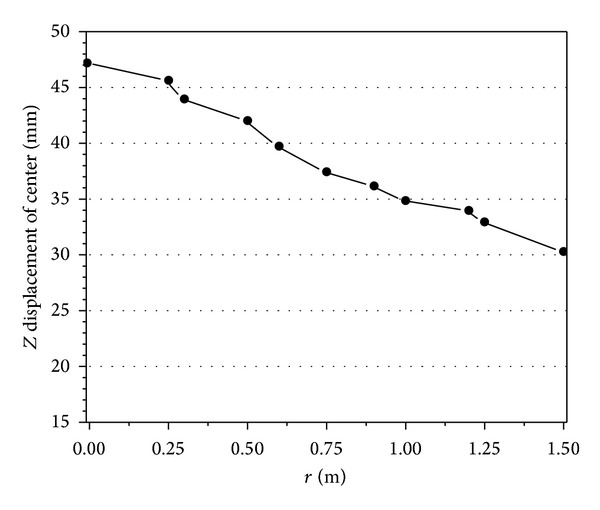
Maximum displacement of slab center under different explosive positions.

**Figure 10:**
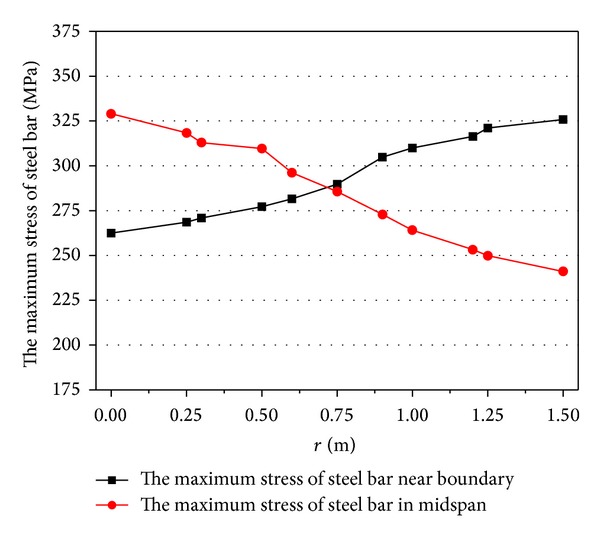
Maximum stress of steel bar under different explosive positions.

In the event of an actual explosion, the explosion may not always occur above the centre of slab. It is necessary to study the dynamic response and failure mode when the explosive position is changed. So keeping the explosive weight *W* = 50 kg and the distance *H* = 5.0 m, the scaled distance is 1.35 m/kg^1/3^, and the peak value of the blast loading exerted on the slab is *P*_max⁡_ = 2.03 MPa. Then the explosive is moved along the *y* axis from centre to boundary. Figures 9 and 10 are the maximum displacement of the centre and the stress of steel bar in the slab, respectively.

These figures showed that when the explosive moved from the slab centre to the boundary (the parameter *r* is the distance from centre to the boundary, *r* = 0 ~ 1.5 m). The maximum displacement of the slab centre is reduced and the stress of the steel bar in tensile zone of slab (in midspan) can be effectively decreased, but the stress of steel bar nearby the boundary is increased. The failure mode changes from flexural failure to the direct-shear failure at the same time. It can be found that although the explosive performance parameters are the same, the failure mode is changed with the explosive position. When the explosive position is moved to the boundary, direct-shear failure may happen easily.

## 4. Damage Assessment of RC Slabs Subject to Blast Loadings

In this section, a new damage assessment criterion is defined and a numerical method to evaluate the damage is proposed in Section 4.1. Then the damage degrees of RC slabs under different explosive cases are evaluated in Section 4.2. Also the effects of negative reinforcement steel bar length and boundary conditions on the antiexplosive ability of RC are analyzed in Section 4.3.

### 4.1. The Criteria of Damage Assessment

The most common damage index for assessing the damage degree is the midspan displacement of slabs or the support rotation, but it is not very effective to evaluate the damage degree if the failure modes are shear failure or shear-bending failure. It needs new criteria to evaluate the damage degree. The new criteria should be proposed based on the following considerations.

1. It should be suitable for evaluating damage degree of RC slabs from all failure modes.
2. It should be related to the global properties of RC slabs.
3. It should be easily obtained for experiments and numerical simulations.

The slab is the important component which suffers direct load in the frame, so we can make a damage assessment criterion by the uniformly distributed load-carrying capacity of the slab as follows:

(6) $$\begin{array}{c} \text{DI}=1-\frac{P_{R}}{P_{O}}, \end{array}$$

where *P*_*O*_ is the original load-carrying capacity and *P*_*R*_ is the residual load-carrying capacity of the slab. Criteria for the various damage levels can be defined [15] as follows: when DI = 0 ~ 0.2, it means that the slab is in a low damage state; DI = 0.2 ~ 0.5, medium damage; DI = 0.5 ~ 0.8, high damage; and DI = 0.8 ~ 1, the structure will be collapsed.

The damage degree of RC two-way bending slab can be obtained by the following steps.

**Figure 11:**
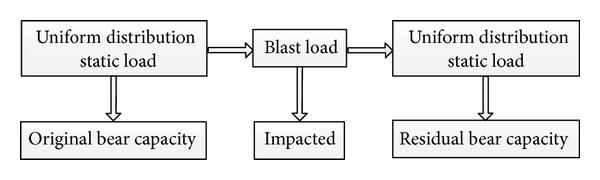
Flowchart of damage assessment program.

1. Establishing the finite element model of RC slab and exerting the uniformly distributed load on the slab. The load is increased slowly from 0 until the slab is destroyed. With the load and the midspan displacement curve, we can get the limit load-carrying capacity of uniformly distributed load.
2. Applying the blast load on the top surface of RC slab and analyzing the dynamic response and damage situation. In order to get the whole response history, it needs a longtime to calculate until the structure gets a new equilibrium state. When the node velocity of the slab reaches 0.1 m/s, we can stop this step and consider the structure at a static equilibrium status.
3. Getting the residual load-carrying capacity of the RC slab through the restarted method. Exerting the uniformly distributed load on the slab again and increasing slowly from 0 until the slab is destroyed again after forcing the node velocity to 0.
4. Calculating the damage degree by (6). The damage assessment program is shown in Figure 11.

**Figure 12:**
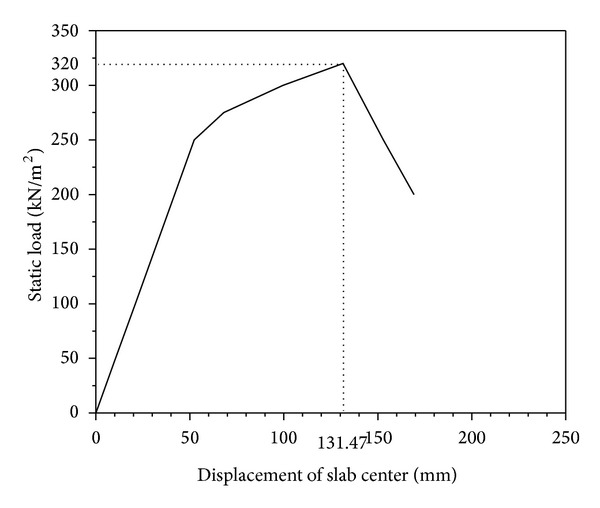
Displacement-static load curve of slab center.

The original load-carrying capacity of RC two-way bending slab is obtained by some numerical simulations.  Figure 12 shows the displacement-pressure curve of the slab centre. When the displacement of center point is at range 0~51 mm, the corresponding pressure value is from 0~250 kN/m^2^, the curve is almost a straight line, and the slab is in an elastic stage. When the displacement is larger than 51 mm, the displacement increases obviously while the pressure has little increase, and the slab is in a plastic stage. When the displacement of slab center is 131.47 mm, the pressure achieves the maximum value. The corresponding original load-carrying capacity *P*_*O*_ is 320 kN/m^2^.

In the same way, the residual load-carrying capacity of the slab is under blast load (*W* = 50 kg, *H* = 5.0 m), that is, *P*_*R*_ is 205.1 kN/m^2^. So DI = 1 − *P*_*R*_/*P*_*O*_ = 1 − 205.1/320 = 0.36, and therefore, the damage of the slab is deemed as in a medium damage state.

### 4.2. Different Load Cases

#### 4.2.1. Different Explosive Weight

**Figure 13:**
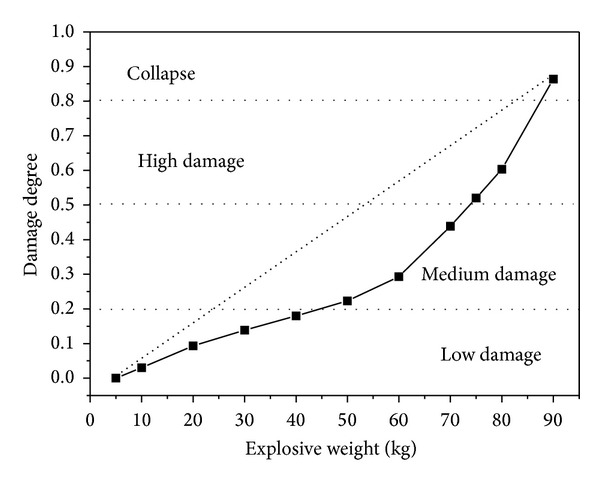
Damage degree of RC slabs under different explosive weights.

The damage degrees of RC two-way bending slab under different explosive weights is shown in Figure 13. When the pressure is tiny with explosive weight *W* = 5 kg, the slab is perfectly intact with the damage degree DI = 0. When the explosive weight is *W* = 10 ~ 40 kg, it is classified as low damage. When *W* = 50 ~ 70 kg, it is classified as medium damage. When *W* = 75 ~ 80 kg, it is classified as high damage. When *W* = 90 kg, the damage degree DI is 0.86, and the slab will be deemed as “collapsed”.

As the transformation law of the maximum displacement, the damage degree of RC slab is increased with explosive weight increases, and the slope of the curve is increasing, too.

#### 4.2.2. Different Explosive Position

**Figure 14:**
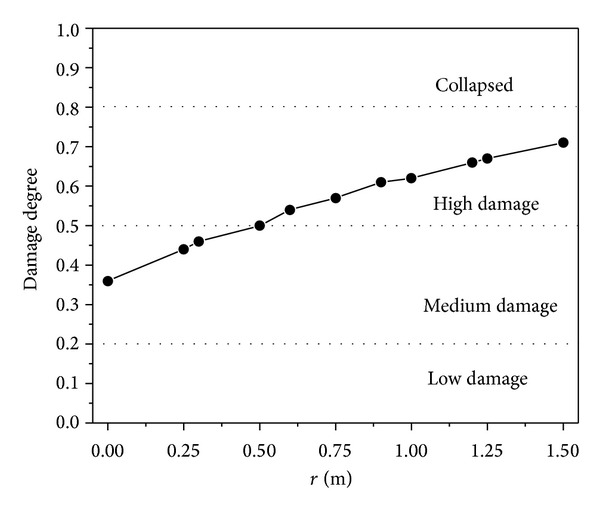
Damage degree of RC slabs under different explosive positions.

From Section 3.3, the failure mode of RC two-way bending slab changes from flexural failure to direct-shear failure as the explosive position is from center to the boundary.  Figure 14 shows the relationship of damage degree and explosive position (*W* = 50 kg and *H* = 5.0 m).

When the distance *r* ≥ 0.5 m, the damage level changed from “medium damage” to “high damage” and the damage degree is increased while the explosive moving to the boundary. The explosion at the corner will cause larger damage. Otherwise, if the effects of corners on blast loadings are considered, the damage will be increased.

### 4.3. Some Design Parameters for Improving the Antiexplosive Ability of RC Slabs

In the two-way bending RC slab design, the boundary conditions will affect the load-carrying capacity directly and the length of negative reinforcement steel bar will affect the seismic design of buildings ability [32]. These parameters may change the antiexplosion ability of the two-way bending RC slab. Based on the above research, we have done some studies on the dynamic response and damage degree of two-way bending RC slab under blast loadings by parameterization analysis.

#### 4.3.1. Length of Negative Reinforcement Steel Bar

As it is defined in the code for design of concrete structures (GB50010-2010) [32], the length of negative reinforcement steel bar from the beam, column, and wall should be at least 1/4 of length of actual span of two-way bending RC slabs for seismic design of buildings.

**Figure 15:**
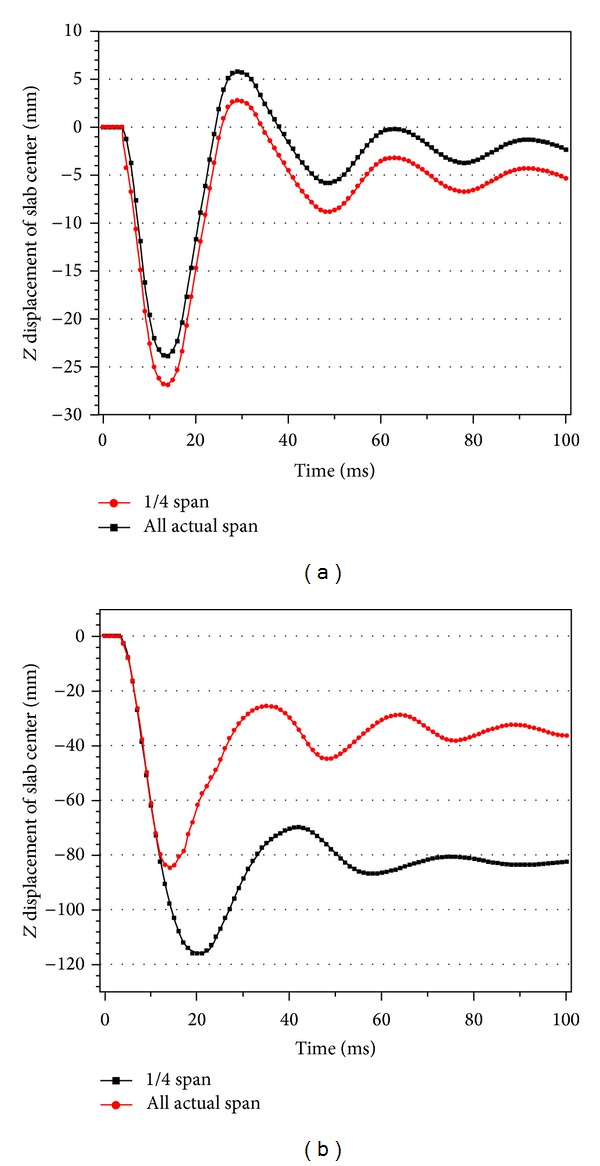
Displacement time histories of RC slab center under different reinforcement types: (a) *W* = 30 kg and (b) *W* = 75 kg.

From Figure 15, when the explosive weight is *W* = 30 kg, there is no significant difference between the two displacement-time histories of slab center. The maximum displacements have only 10% change. When the explosive weight is *W* = 75 kg, the displacement-time history changes a lot. The maximum displacement and residual deformation of the slab are reduced obviously as the length of negative reinforcement is actual span. The damage degree of two-way bending RC slabs with different negative reinforcement length is listed in Table 2. The limit load-carry capacities of the slab have not changed so much, and the difference between 1/4 and actual span length of negative reinforcement is only 6%. The damage degree of the slab is 0.19 for 1/4 of length and 0.17 for actual span subject to blast loadings caused by explosive weight *W* = 30 kg, and the damage states are in the low damage. But for larger explosive weight *W* = 75 kg, the damage situation changes obviously. The slab with actual span negative reinforcement is in the high damage while the slab with 1/4 of length of span is collapsed; the antiexplosion ability is improved by increasing the length of negative reinforcement. That is because the slab will vibrate up and down under the blast loadings and the bending resistance will increase with the length of negative reinforcement. So the antiexplosion ability is increased.

#### 4.3.2. Different Boundary Conditions

**Figure 16:**
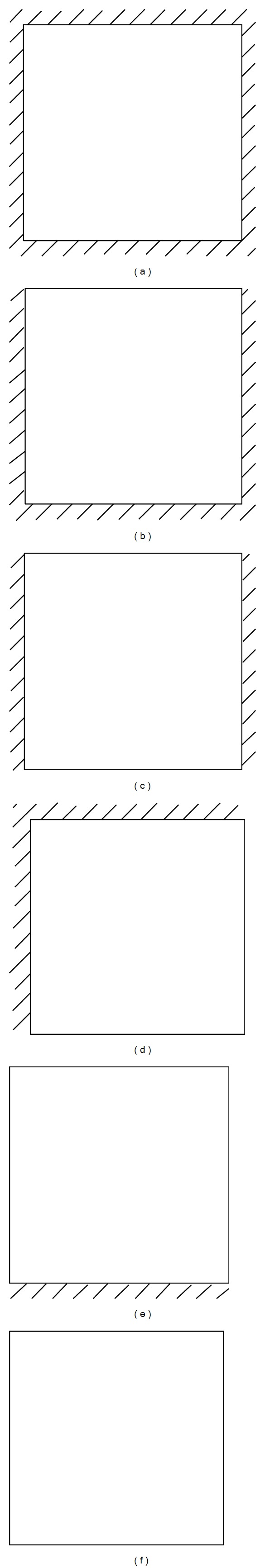
Sketch under different boundary conditions: (a) four edges are clamped, (b) three edges clamped and one edge simply supported, (c) opposite edges clamped and others simply supported, (d) adjacent edges clamped and others simply supported, (e) three edges simply supported and one clamped, and (f) four edges simply supported.

The actual boundary condition of two-way bending RC slab is between simply and clamped supported, and it can always be treated as simple or clamped supports. The simply supported slab can only pass the force of horizontal and vertical directions, but cannot pass the bending moment. The clamped-supported slab can pass not only the horizontal and vertical force well but also the bending moment. The most common support forms of two-way bending RC slab [32] are supported by four edges as shown in Figure 16. In the numerical simulations, the constraint of RC slab is achieved by changing the support of rigid beam.

**Figure 17:**
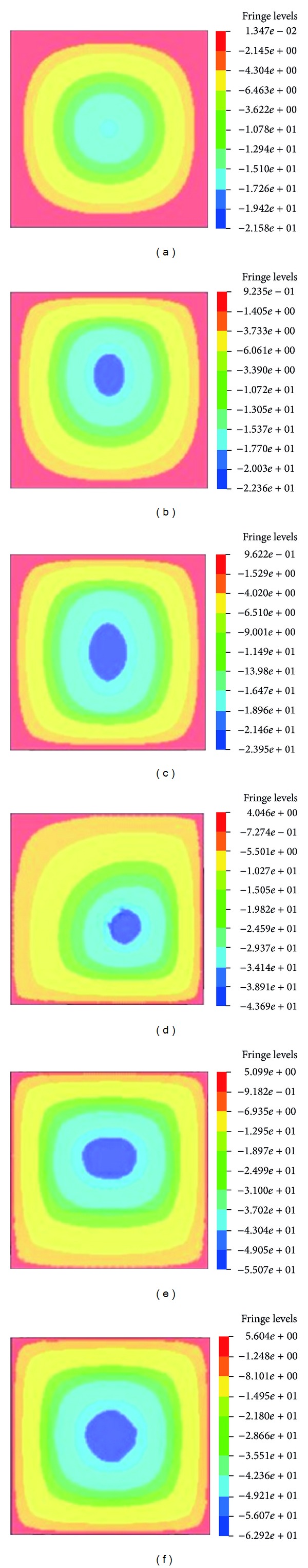
Displacement contours of RC slabs under different boundary conditions.

In order to analyze the effects of the boundary condition, different boundaries with upper models and the blast case of *W* = 30 kg and *H* = 5.0 m are used.  Figure 17 shows the displacement contours of the moment when the vertical displacement achieves the maximum value. Although the explosive position is over the centre of the slab, the maximum displacements did not appear in the slab centre and the maximum displacement is moved to the simply supported side. The constraint effect is weakening with the increase of the simply supported number, and the maximum displacement of slab centre is increased. It is clear that the antiexplosion ability of two-way bending RC slab will change with the boundary changes.

The damage degrees under different boundary conditions are given in Table 3. When the four edges are clamped, the damage degree DI is 0.17, which is in the low damage. When there are one or two simply supported edges, the damage levels are in the medium damage. When the number of simply supported edges is three or four, the slab is in the high damage. At the same time, the limit load-carrying capacity is reduced with the number increase of simply supported edges.

## 5. Conclusions

In this study, a damage degree assessment criterion is defined based on the residual uniformly distributed load-carrying capacity of a two-way bending RC slab. Using this criterion, the damage degrees of the slab under different explosion cases and design parameters are analyzed. The main results are listed as follows.

1. The damage degree assessment criterion based on the residual uniformly distributed load-carry capacity presents a new index to evaluate the damage degree of the two-way bending RC slab; it can be applied to all failure modes.
2. Subjected to blast loadings, the damage degree is directly affected by the explosive weights and positions. The damage degree would increase when the explosive weight is increased or the explosive position is moved from centre to the boundary.
3. The boundary conditions and the length of negative reinforcement steel bar can directly affect the antiexplosion ability of the slab. For the two-way bending RC slab antiexplosion design, it is better to reduce the number of simply supported edges and layout the negative reinforcement steel bar along the actual span.

## Figures and Tables

**Table 1:** Maximum displacement of RC slab under different explosive charges.

Explosive weight (kg)	Distance (m)	Scaled distance (m/kg^1/3^)	Peak value of blast load (MPa)	Maximum displacement of centre point (mm)
5	5	2.92	0.23	3.46
10		2.32	0.42	6.99
30		1.61	1.22	19.96
50		1.35	2.03	47.21
75		1.18	3.03	84.28
100		1.08	4.02	158.08

**Table 2:** Damage degree of RC slab under different reinforcement models.

Length of negative reinforcement	Original bear capacity (kN/m^2^)	Damage degree	
		*W* = 30 kg	*W* = 75 kg
1/4	299	0.19	0.83
All actual span	320	0.17	0.57

**Table 3:** Damage degree of RC slab under different boundary conditions.

Boundary condition	Original bear capacity (kN/m^2^)	Residual bear capacity (kN/m^2^)	Damage degree	Damage level
Case (a)	320	256.6	0.17	Low damage
Case (b)	292	226.2	0.22	Medium damage
Case (c)	279	193.2	0.31	Medium damage
Case (d)	253	142.5	0.43	Medium damage
Case (e)	231	96.6	0.58	High damage
Case (f)	197	54	0.73	High damage
